# The impact of community-based health insurance on universal health coverage in Ethiopia: a systematic review and meta-analysis

**DOI:** 10.1080/16549716.2023.2189764

**Published:** 2023-03-22

**Authors:** Ewunetie Mekashaw Bayked, Husien Nurahmed Toleha, Seble Zewdu Kebede, Birhanu Demeke Workneh, Mesfin Haile Kahissay

**Affiliations:** a Department of Pharmacy, College of Medicine and Health Sciences (CMHS), Wollo University, Dessie, Ethiopia; b Department of Pharmacy, Dessie College of Health Sciences (DCHS), Dessie, Ethiopia; c Department of Pharmaceutics and Social Pharmacy, School of Pharmacy, College of Health Sciences, Addis Ababa University, Addis Ababa, Ethiopia

**Keywords:** Financial risk protection, health-related quality of life, healthcare seeking behavior, health service quality, population coverage

## Abstract

**Background:**

Ideally health insurance aims to provide financial security, promote social inclusion, and ensure equitable access to quality healthcare services for all households. Community-based health insurance has been operating in Ethiopia since 2011. However, its nationwide impact on universal health coverage has not yet been evaluated despite several studies being conducted.

**Objective:**

We evaluated the impact of Ethiopia’s community-based health insurance (2012–2021) on universal health coverage.

**Methods:**

On 27 August 2022, searches were conducted in Scopus, Hinari, PubMed, Google Scholar, and Semantic Scholar. Twenty-three studies were included. We used the Joana Briggs Institute checklists to assess the risk of bias. We included cross-sectional and mixed studies with low and medium risk. The data were processed in Microsoft Excel and analyzed using RevMan-5. The impact was measured first on insured households and then on insured versus uninsured households. We used a random model to measure the effect estimates (odds ratios) with a *p* value < 0.05 and a 95% CI.

**Results:**

The universal health coverage provided by the scheme was 45.6% (OR = 1.92, 95% CI: 1.44–2.58). Being a member of the scheme increased universal health coverage by 24.8%. The healthcare service utilization of the beneficiaries was 64.5% (OR = 1.95, 95% CI: 1.29–2.93). The scheme reduced catastrophic health expenditure by 79.4% (OR = 4.99, 95% CI: 1.27–19.67). It yielded a 92% (OR = 11.58, 95% CI: 8.12–16.51) perception of health service quality. The health-related quality of life provided by it was 63% (OR = 1.71, 95% CI: 1.50–1.94). Its population coverage was 40.1% (OR = 0.64, 95% CI: 0.41–1.02).

**Conclusion:**

Although the scheme had positive impacts on health service issues by reducing catastrophic costs, the low universal health coverage on a limited population indicates that Ethiopia should move to a broader national scheme that covers the entire population.

## Introduction

Health is an integral part of the Sustainable Development Goals (SDGs) [1]. Universal health coverage (UHC) is the target of SDG-3 [2]. In particular, the SDG 3.8 target aims to achieve UHC, including financial risk protection (FRP), access to quality essential health services, and safe medicines and vaccines for all [1]. The UHC is therefore defined in a way that ensures that all people have access to quality health services while avoiding financial hardship due to their use. The core concepts of UHC are population coverage (PC), health service delivery, and out-of-pocket (OOP) expenses [3,4]. Moreover, without UHC, SDG 1 may be jeopardized, as health costs impoverish nearly 90 million people each year. On the other hand, access to quality and affordable primary health care (PHC) is the cornerstone of UHC [1].

Since the Alma-Ata Declaration of 1978, Ethiopia has made various efforts to provide PHC to its citizens. It is the most populous landlocked country in Africa and the second-most populous nation on this continent. The Ethiopian healthcare system is funded by loans and donations (46.8%), the government (16.5%), individual contributions (35.8%), and others (0.9%) [5]. However, effective resource mobilization through health insurance, rather than loans and donations, is preferred for achieving UHC. Because health insurance protects beneficiaries from unanticipated and often catastrophic healthcare expenditure [6], the lack of effective health insurance programs is 65 a major obstacle to achieving UHC [7].

**Figure 1.**
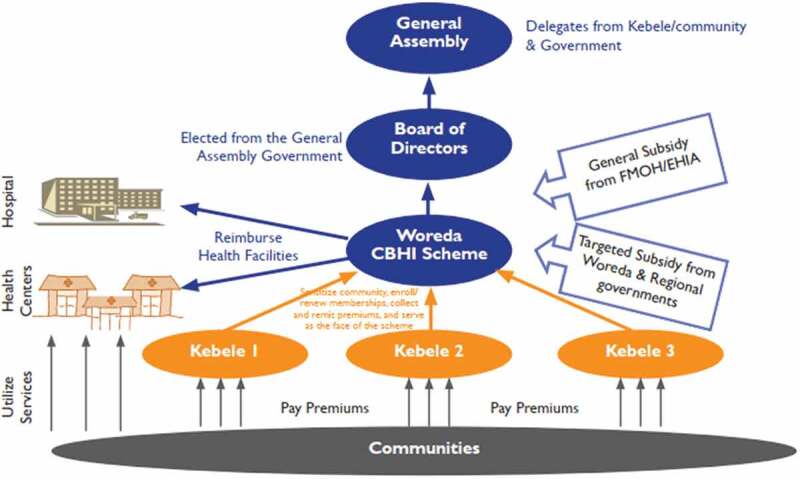
Flow of finance, governance, and organizational structure of CBHI schemes, Ethiopia [11].

National health insurance (NHI), social health insurance (SHI), private health insurance (PHI), and community-based health insurance (CBHI) are the four main categories of health insurance programs [8]. Currently, six African countries – Rwanda, Tanzania, Mali, Ghana, Senegal, and Ethiopia – are implementing CBHI as a mechanism to achieve UHC [9]. CBHI aims to improve access to quality health services for low-income rural households not covered by formal insurance [10]. It is a non-profit private health insurance program based on the concept of mutual aid in rural and underdeveloped communities. As depicted in Figure 1, it combines premium contributions from members into a group fund that is run by the members [8].

Since 2010, the Ethiopian government has been working to introduce a CBHI for the informal sector as a means to achieve UHC [12]. Implementation started in 2011 [9,11]. Thirteen rural districts in the country’s four major regions – Tigray, Amhara, Oromia, and the Southern Nations, Nationalities, and Peoples Region (SNNPR) – were the first to implement the program [13]. Based on the promising results of the pilot implementation, the scale-up started in 2015 [14] as a means to achieve UHC [11], which is the objective of the Second Health Sector Transformation Plan (HSTP-II) of Ethiopia [15].

Despite widespread optimism about pooling resources to cover healthcare costs, CBHI has a limited impact on ensuring participants have access to the healthcare and financial security they need. This indicates that participation is also low, usually leaving the poorest behind. The scheme appeared to have a limited role in helping countries transition to UHC [16]. Accordingly, it was important to evaluate the impact of CBHI over time.

Although CBHI has been operating in Ethiopia since 2011, the impact and contribution to UHC have not yet been evaluated. The objective of this systematic review and meta-analysis was to evaluate the impact of CBHI in Ethiopia on the country’s progress towards UHC (2012–2021).

## Methods

### Registration and protocol

The protocol for this review was registered at PROSPERO with ID CRD42022355972. Necessary amendments were made to the protocol during the review process. As provided in Supplementary File 1, the ‘Preferred Reporting Items for Systematic Reviews and Meta-Analyses (PRISMA) 2020 statement: an updated guideline for reporting systematic reviews’ was used as the framework for the review [17]. In accordance with PRISMA 2020, we discussed the literature selection procedures, while the PRISMA 2009 flow chart was used for the pictorial representation [18].

### Eligibility criteria

All analytical, prevalent, and retrospective cross-sectional studies and mixed study designs were considered. To estimate the current PC, recent systematic reviews and national studies conducted within the last three years and reporting pooled data were included rather than individual studies. All published studies in English conducted from 2012 to 2021, both in communities and institutions, on the impact of CBHI on UHC in Ethiopia’s informal sector were considered. The following study parameters were also used to decide which studies to include: outcome variables, population (study units), year of the study, context (regions), sample size, and response rate. Moreover, as stated above, review articles published after 2021 were taken into consideration if the original articles they included were within the scope of the review.

All other studies with incomplete data, conducted before the CBHI’s first-year implementation report (2012) and after 2021, and with a high risk of bias were excluded. In addition, if a study had both published and unpublished copies with identical reports, the unpublished copies were excluded. Furthermore, studies published in multiple journals were considered duplicates, and the most recently published studies were selected for inclusion in the review. In general, studies reporting the desired outcome variables were first selected to be included in the systematic review. Then, from the studies eligible for the systematic review, quantitative studies that reported comparators – intervention (insured households) and control groups (uninsured households) – were selected for the meta-analysis.

### Information sources and search strategy

Database searches were performed on Scopus, Research4Life (Hinari), PubMed, Google Scholar, and Semantic Scholar on 27 August 2022 (Supplementary File 2). PubMed and Hinari resources were searched manually. However, Scopus, Google Scholar, and Semantic Scholar were searched using the ‘Perish or Publish’ database searching tool, version 8 [19]. Registries such as the Ethiopian Health Insurance Service (EHIS) and the general web were also searched for additional information. The databases were searched using text words and indexed terms such as ‘community-based health insurance,’ ‘impact,’ ‘effect,’ ‘role,’ and ‘Ethiopia.’ Additional filters were also used: year of study, publication year, content type, discipline, and language. Reference lists of studies meeting the inclusion criteria were searched to find more relevant studies.

### Selection process

After duplicates and irrelevant studies had been excluded using Zotero reference manager version 6, two reviewers, EMB and HNT, independently screened the included studies. The selection of studies has been carefully screened by these two researchers. First, the articles were refined by their title and abstract; second, by full-text revision by these authors, independently and finally together, until reaching consensus. When disagreements occurred, a third reviewer was contacted to resolve the disagreement. Then, as stated under the eligibility criteria and study risk of bias assessment sections above and below, respectively, all studies that fulfiled the eligibility criteria and had a score of low or medium risk of bias were included.

### Data collection process

A Microsoft Excel spreadsheet was prepared, tested, adjusted, and used for data extraction. The outcome variables – population (study units), year of study, context, sample size, response rate, and proportions – were extracted by the Excel spreadsheet. Two reviewers, EMB and HNT, independently extracted the data, compared conclusions, and reached agreement. If not, a third reviewer was invited to review with these two to reach consensus. Moreover, we contacted the study authors to collect the missing information.

### Data items

The main outcome of this review was the impact of CBHI on UHC, which includes three main concepts: PC, range of health services provided, and OOP expenditure – FRP, cost of care (COC), or catastrophic healthcare expenditure (CHE). In addition to these primary outcome variables, health service utilization (HSU) or health-seeking behavior (HSB), access and health service quality (HSQ), health-related quality of life (HRQoL), and household economic welfare were extracted. These concepts are the key indicators of UHC, and their definitions are provided as follows:

- **CHE**: It is the primary indicator of FRP and can be defined based on a number of scenarios. Based on the budget share approach, it refers to the “number of people spending 25% or more of their total expenditure on OOP health expenditures.” According to the capacity to pay based on subsistence needs, it refers to the “number of people spending 40% or more of their capacity to pay on OOP.” From the perspective of the capacity to pay based on food expenditure, it refers to the “number of people spending 40% or more of their non-food expenditures on OOP” [20].
- **COC**: This **r**efers to the “costs for individuals directly or indirectly incurred by the provision of health-care goods and services, aimed at maintaining or recovering the health of a person” [21].
- **FRP**: It is the “access of households to needed healthcare services without experiencing undue financial hardship” [22]. It is a key component of UHC [23].
- **HRQoL**: It refers to “reports of patients or individuals regarding functioning and well-being in the physical, mental, and social domains of life” [24].
- **HSB**: It refers to “any action undertaken by individuals who perceive themselves to have a health problem or to be ill for the purpose of finding an appropriate remedy” [25].
- **HSQ**: It is “the degree to which health services for individuals and populations increase the likelihood of desired health outcomes and are consistent with current professional knowledge.” “It spans both curative and preventive care and facility- and community-based care for individuals and populations” [26].
- **HSU**: It refers to “how much health care people use, the types of health care they use, and the timing of that care” [27].
- **OOP health expenditure**: This refers to the “direct expenses by individuals to health care providers, excluding any prepayments for health services, such as taxes, insurance premiums, or contributions” [28].
- **PC**: It is the “share of the population covered for a defined set of health care goods and services under public programs and through private health insurance” [29]. Here, it refers to the percentage of the population covered by the CBHI scheme.

### Study risk of bias assessment

The risk of bias in the included studies was independently assessed by two reviewers, EMB and HNT, using tools developed by the Joanna Briggs Institute (JBI). The bias was assessed on: criteria for inclusion in the sample, descriptions of study subjects and settings, validity and reliability of measurement, confounding and strategies to deal with it, and appropriateness of the outcome measure. The JBI’s tools with 8, 10, and 11 items were used to assess cross-sectional, case-control, and review articles, respectively. As a result, cross-sectional studies and mixed studies with a cross-sectional design score of 7 or higher were labeled as low risk, 5–6 medium risk, and 4 or lower high risk. However, for the case-control and systematic review studies, scores of 6 and below, 7–9, and greater than 9 were rated as high risk, medium risk, and low risk, respectively. After manual appraisal, the risk of bias was summarized using RevMan 5.4.1. Then, those studies with low and medium risk were included in the study. Any inconsistencies were resolved by discussion and involving a third reviewer, as necessary.

### Effect measures

Prevalence, proportions, inverse variance (IV), and odds ratios (ORs) were calculated for each study. For summary effects, X^2^, *z* value, *p* value with a 95% CI, and odds ratios were computed.

### Synthesis methods

For the qualitative synthesis, we used thematic strategies to conceptually categorize the outcome variables. Based on the qualitative synthesis, preliminary effect measures were computed for the quantitative synthesis using a Microsoft Excel spreadsheet. First, a population group analysis for the outcome variables was performed using only insured households. Second, two-population group analyses comparing households with insurance versus those without it were performed. The results of the PC, HSU, FRP, HRQoL, and HSQ were used to calculate the pooled UHC. However, since uninsured households are not enrolled in the scheme, the PC was excluded from the two-population group.

We used RevMan 5.4.1 to calculate the pooled effect estimates, the ORs using a random method. Sub-group analyses were conducted to compare the effect estimates across studies on the outcome variables. The level of overall statistical significance was determined at a *p* value less than 0.05 with a 95% CI.

### Reporting bias assessment

Reporting bias was assessed by considering whether the studies were published or not. It was also examined by the publication years of the studies. For those studies with incomplete or missing data, the study authors were contacted. The studies with incomplete data were excluded.

### Certainty assessment

The I^2^-statistic was used to assess heterogeneity between studies. The influence of each study on the overall meta-analysis was measured using IV (percentage of weight). Funnel plots were used to examine potential inter-study bias (publication bias). Sensitivity analysis was performed by unchecking studies with small sample sizes (*n* < 200).

## Results

### Study selection

**Figure 2.**
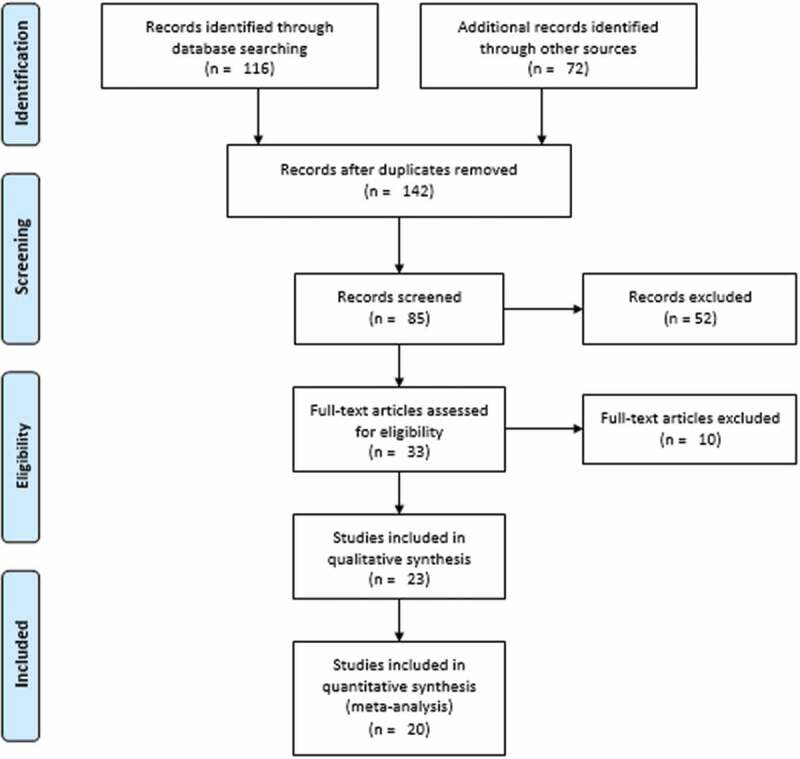
PRISMA flow diagram showing the selection processes of the included studies.

In total, 188 resources were identified (Figure 2). One hundred and sixteen of them were identified from databases: Scopus (*n* = 10), Hinari (*n* = 31), PubMed (*n* = 40), Google Scholar (*n* = 15), and Semantic Scholar (*n* = 20). The rest were identified from other sources: websites (*n* = 31), organizations (*n* = 11), registries (*n* = 23), and citation searches (*n* = 7). One hundred forty-two records were identified after duplicates (*n* = 46) were removed. After excluding 57 studies based on relevance, 85 studies were screened for title and abstract evaluation. Through title and abstract review, 33 records were chosen to be eligible for full text evaluation. Due to incomplete data (*n* = 6) [12, 30–34], publication in more than one journal or reporting identical findings (*n* = 2) [35–38], and a high risk of bias (*n* = 2) [39,40], a total of 10 publications were eliminated through the full text evaluation. Finally, 23 studies were included in the qualitative synthesis. From these, 20 records were included in the meta-analysis for one-population group (insured households only). For the two-population groups, insured (intervention) versus uninsured (control), 13 studies were included.

### Study characteristics

From the total number of studies (*n* = 23) included in the systematic review, more than half (*n* = 12) were conducted in the Amhara region. The rest were conducted in Addis Ababa (*n* = 1), SNNPR (*n* = 2), Oromia (*n* = 1), and the national context (*n* = 7). The individual studies were assessed for study design, area (context), year of study, sample size, non-response rate or response rate, and main outcomes. In total, the sample population of all the included studies was 48,716, of which 48,625 (99.8%) were found to be actual participants. The summary results of the individual study characteristics are presented in Table 1.

**Table 1.** Characteristics of the individual included studies, Ethiopia (*n = 23*), 2022.

Study ID	Design	Area	Year	SS	RR	Main Outcome
(Seid and Ahmed, 2021)	Cross-sectional	National	2016	4278	4278	HSU
(Atnafu and Gebremedhin, 2020)	Cross-sectional	Amhara	2017	226	226	HSU
(Segahu, 2018)	Cross-sectional	Oromia	2018	280	270	HSU & access
(Tiruneh et al., 2018)	Case-control	Amhara	2014	318	318	HSB
(Simieneh et al., 2021)	Cross-sectional	Amhara	2016	410	410	HSB
(Tilahun et al., 2018)	Cross-sectional	Amhara	2016	652	594	HSU
(Alemayehu et al., 2022)	Cross-sectional	National	2020	4238	4238	HSU & FRP
(Mebratie et al., 2019)	Survey	National	2011–13	1569	1569	HSU & COC
(Demissie and Negeri, 2020)	Mixed	SNNPR	2017	405	405	HSU
(Jembere, 2018a)	Mixed	Amhara	2017	344	344	Access, HSU & HSQ
(Mekonen et al., 2018)	Cross-sectional	Amhara	2016	454	454	FRP (CHE)
(Moyehodie et al., 2022)	Cross-sectional	Amhara	-	619	619	HSU
(Engida, 2019)	Cross-sectional	Amhara	2019	634	634	HSU
(Abenet et al., 2019)	Mixed	Amhara	2018	376	376	HSU
(Gebru and Lentiro, 2018)	Cross-sectional	SNNPR	2017	1964	1955	HRQoL
(Asfaw et al., 2022)	Cross-sectional	Amhara	-	531	531	Household’s Welfare
(Tefera et al., 2021)	Mixed	National	2019	556	556	HSQ
(Jembere, 2018b)	Mixed	Amhara	2017	344	344	FRP & HDB
(Dagnaw et al., 2022)	Cross-sectional	Amhara	2021	658	648	HSU
(Habte et al., 2022)	SR & MA	National	2022	8418	8418	PC
(Tahir et al., 2022)	SR & MA	National	2022	12127	12127	PC
(Terefe et al., 2022)	Cross-sectional	National	2019	8663	8663	PC
(Girmay and Reta, 2022)	Cross-sectional	Addis Ababa	2021	652	648	HSU

MA: Meta-Analysis; SNNPR: Southern Nations, Nationalities and Peoples Region; RR: Response Rate; SR: Systematic Review; SS: Sample Size.

### Risk of bias in studies

**Figure 3.**
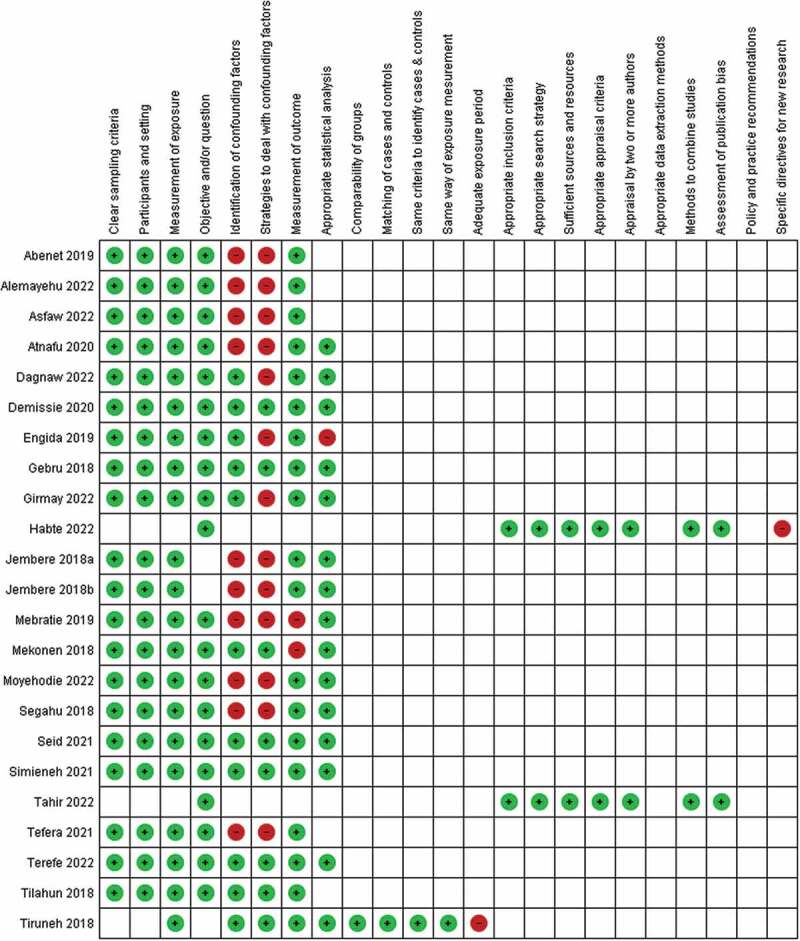
Risk of bias assessment summary: red = high risk; green = low risk; and unfilled = unclear risk.

After the risk of bias for the included studies was assessed using the JBI critical appraisal tools, those studies with a low or medium risk were included in the review. The summary of the risk of bias assessment for each study has been given in Figure 3. The rating of the included studies is provided in Table 2.

**Table 2.** A summary of the rating and ranking of the included studies.

Study ID	Score		Risk
	Tally	Percentage	
1. (Seid and Ahmed, 2021)	8/8	100	Low
2. (Atnafu and Gebremedhin, 2020)	6/8	75	Medium
3. (Segahu, 2018)	6/8	75	Medium
4. (Tiruneh et al., 2018)	9/10	90	Medium
5. (Simieneh et al., 2021)	8/8	100	Low
6. (Tilahun et al., 2018)	8/8	100	Low
7. (Alemayehu et al., 2022)	5/8	62.5	Medium
8. (Mebratie et al., 2019)	6/8	75	Medium
9. (Demissie and Negeri, 2020)	8/8	100	Low
10. (Jembere, 2018a)	5/8	62.5	Medium
11. (Mekonen et al., 2018)	7/8	87.5	Low
12. (Moyehodie et al., 2022)	5/8	62.5	Medium
13. (Engida, 2019)	6/8	75	Medium
14. (Abenet et al., 2019)	5/8	62.5	Medium
15. (Gebru and Lentiro, 2018)	8/8	100	Low
16. (Asfaw et al., 2022)	5/8	62.5	Medium
17. (Tefera et al., 2021)	5/8	62.5	Medium
18. (Jembere, 2018b)	5/8	62.5	Medium
19. (Dagnaw et al., 2022)	7/8	87.5	Low
20. (Habte et al., 2022)	7/11	63.63	Medium
21. (Tahir et al., 2022)	8/11	72.73	Medium
22. (Terefe et al., 2022)	8/8	100	Low
23. (Girmay and Reta, 2022)	7/8	87.5	Low

### Results of individual studies

#### Qualitative result

Based on the concepts of UHC, the qualitative findings of the included studies were thematized into five categories.

- **PC**: The PC of the CBHI has gradually been expanded [38,41,42].
- **HSU**: CBHI improved HSU [36,43–56], such as antenatal care (ANC) visits [43]; child healthcare visits [44,47]; seeking treatment for malaria [46]; in-patient [36] and outpatient [49,50] attendances; frequency of visits [49,53]; and family planning [53].
- **FRP**: CBHI has improved FRP [45,48,49,53,55,57,58]. As such, it reduced OOP [55], CHE [45,57,58], and COC [49]. In doing so, it improved household welfare [59].
- **HSQ**: CBHI has contributed to the provision of HSQ [55,60]. It improved diagnostic test capacity, the availability of tracer drugs, provider interpersonal communication, and service quality standards. The scheme increased the accountability of health facilities in CBHI districts because they promised to provide quality services using the CBHI premium collected at the beginning of the year from all enrolled households [60]. It also improved access to modern healthcare services [45,55].
- **HRQoL**: CBHI has promisingly improved HRQoL [61].

#### Quantitative result

Quantitative data were extracted from 20 of the 23 studies included in the review.

#### One-population group (insured)

The quantitative data for the one-population group is presented in Table 3. The pooled UHC by CBHI was found to be 45.6%. Regarding PC, according to the three national studies included in this review, 40.10% of the households were found to be covered by the scheme, with the lowest, medium, and highest coverages being 28% [38], 45% [42], and 45.5% [41], respectively. Coming to the impact of CBHI on HSU, the pooled report of the 13 included studies showed that the HSU among CBHI members was 64.5%. The lowest and highest rates were reported in SNNPR (19.3%) [50] and Amhara (95.5%) [44], respectively. The pooled non-exposure to CHE in receiving health services was found to be 79.4%. The lower and higher percentages of reports regarding FRP were 71.5% [58] and 91.1% [57]. The HRQoL and HSQ were 63% [61] and 92.1% [60], respectively.

**Table 3.** The prevalence of the quantitative outcomes of the one-population group (insured), Ethiopia (*n* = 20), 2022.

Study ID	No. participants	Events	Prevalence (%)	Region
**Population coverage (PC)**				
(Habte et al., 2022)	8418	3830	45.5	National
(Tahir et al., 2022)	12127	5457	45	National
(Terefe et al., 2022)	8663	2426	28	National
**Total**	29208	11713	40.10	Pooled
**Health service utilization (HSU)**				
(Moyehodie et al., 2022)	619	511	82.6	Amhara
(Engida, 2019)	634	448	70.7	Amhara
(Segahu, 2018)	126	114	90.48	Oromia
(Tilahun et al., 2018)	297	150	50.51	Amhara
(Tiruneh et al., 2018)	144	121	84.03	Amhara
(Mebratie et al., 2019)	569	216	37.96	National
(Atnafu and Gebremedhin, 2020)	111	106	95.50	Amhara
(Demissie and Negeri, 2020)	135	26	19.26	SNNPR
(Seid and Ahmed, 2021)	199	73	36.68	National
(Simieneh et al., 2021)	205	126	61.46	Amhara
(Alemayehu et al., 2022)	1586	1110	69.99	National
(Dagnaw et al., 2022)	329	223	67.78	Amhara
(Girmay and Reta, 2022)	648	389	60	Addis Ababa
**Total**	5602	3613	64.50	Pooled
**Financial risk protection (FRP)**				
(Jembere, 2018b)	334	239	71.5	Amhara
(Mekonen et al., 2018)	224	204	91.07	Amhara
**Total**	558	443	79.39	Pooled
**Health-related quality of life (HRQoL)**				
(Gebru and Lentiro, 2018)	982	619	63.03	SNNPR
**Health service quality (HSQ)**				
(Tefera et al., 2021)	415	382	92.05	National
**Overall (UHC)**	**36765**	**16,770**	**45.61**	

#### Two-population group (insured vs uninsured)

From all included studies, we found 13 studies reporting comparative data for two-population groups (insured vs. uninsured), as shown in Table 4. The pooled HSU was 61.2% and 36.5% among insured and uninsured households, respectively. The lowest HSU among the insured and uninsured households was 19.3% [50] and 14.4% [50], respectively. The highest HSU among insured and uninsured households were 95.5% and 76.5% [44], respectively. The FRP [57], HRQoL [61], and HSQ [60] among the insured households were found to be 91.1%, 63%, and 92.1%, whereas the FRP [57], HRQoL [61], and HSQ [60] for the comparator group (uninsured households) were 69.1%, 59%, and 87.2%, respectively.

**Table 4.** The prevalence of the quantitative outcomes of the two-population group (insured versus uninsured), Ethiopia (*n* = 13), 2022.

		Insured			Uninsured		
Study ID	Participants	Total	Event (%)	Non-event	Total	Event (%)	Non-event
**Health service utilization (HSU)**							
(Segahu, 2018)	270	126	114 (90.48)	12	144	72 (50.0)	72
(Tilahun et al., 2018)	594	297	150 (50.51)	147	297	87 (29.29)	210
(Tiruneh et al., 2018)	318	144	121 (84.03)	23	174	38 (21.84)	136
(Mebratie et al., 2019)	1185	569	216 (37.96)	353	616	240 (38.96)	376
(Atnafu and Gebremedhin, 2020)	226	111	106 (95.50)	5	115	88 (76.52)	27
(Demissie and Negeri, 2020)	405	135	26 (19.26)	109	270	39 (14.44)	231
(Seid and Ahmed, 2021)	4278	199	73 (36.68)	126	4079	958 (23.49)	3121
(Simieneh et al., 2021)	410	205	126 (61.46)	79	205	74 (36.10)	131
(Alemayehu et al., 2022)	3449	1586	1110 (69.99)	476	1863	1248 (66.99)	615
(Dagnaw et al., 2022)	658	329	223 (67.78)	106	329	111 (33.74)	218
**Total**	**11793**	**3701**	**2265 (61.20)**	**1436**	**8092**	**2955 (36.52)**	**5137**
**Financial risk protection (FRP)**							
(Mekonen et al., 2018)	454	224	204 (91.07)	20	230	159 (69.13)	71
**Health-related quality of life (HRQoL)**							
(Gebru and Lentiro, 2018)	1964	982	619 (63.03)	363	982	579 (58.96)	403
**Healthcare service quality (HSQ)**							
(Tefera et al., 2021)	556	415	382 (92.05)	33	141	123 (87.23)	18
**Overall**	**14,767**	**5322**	**3470 (65.20)**	**1852**	**9445**	**3816 (40.40)**	**5629**

### Results of synthesis

#### One-population group (insured)

**Figure 4.**
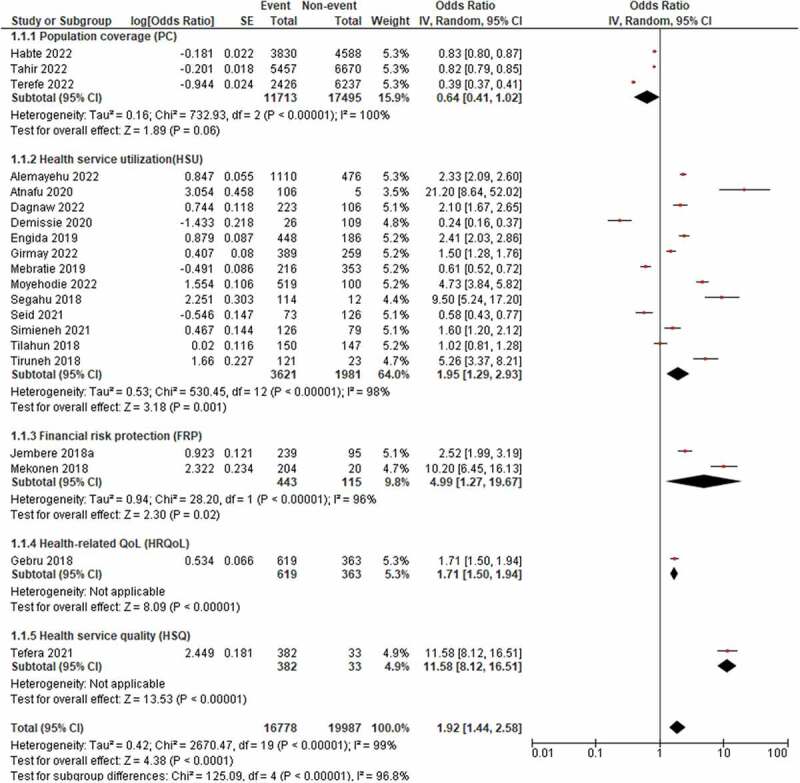
The forest plot for the one-population group (insured), Ethiopia, 2022.

The Mantel-Haenszel statistics were used to calculate the pooled OR. Accordingly, as stated in Table 5 and Figure 4, the test for the overall effect was found to be significant (*P* = 0.0001), with 4.38 standard deviations above the mean. The combined data revealed that the use of CBHI increased the likelihood of UHC by 1.92 times (OR: 1.92, 95% CI: 1.44–2.58). The pooled effect of PC by CBHI was not found to be significant (*P* = 0.06), with 1.89 standard deviations above the mean. The probability of PC by CBHI was found to be 36% less likely (OR = 0.64, 95% CI: 0.41–1.02). However, when a study was unchecked for sensitivity analysis [38], the pooled result of the PC was significant (*P* < 0.00001, OR = 0.82, 95% CI: 0.80–0.85), with 13.85 standard deviations above the mean. Even in this case, however, the probability of PC by CBHI was 18% less likely. Using CBHI was found to reduce the probability of being exposed to CHE by 4.99 times (OR = 4.99, 95% CI: 1.27–19.67). Regarding the impact of CBHI on HSU, the pooled effect revealed that using CBHI was shown to increase the probability of HSU by a factor of 1.95 (OR = 1.95, 95% CI: 1.29–2.93). CBHI was found to increase the probability of HSQ by 11.58 times (OR = 11.58, 95% CI: 8.12–16.51). The probability of HRQoL was found to be increased by 1.71 times when using CBHI (OR = 1.71, 95% CI: 1.50–1.94).

**Table 5.** The pooled result of the impact of CBHI on UHC for the one-population group (insured), Ethiopia (*n* = 20), 2022.

Outcome	Studies	Participants	Events	%	Statistical Method	Effect Estimate
1. PC	3	29208	11713	40.10	Odds Ratio (IV, Random, 95% CI)	0.64 [0.41, 1.02]
2. HSU	13	5602	3613	64.50	Odds Ratio (IV, Random, 95% CI)	1.95 [1.29, 2.93]
3. FRP	2	558	443	79.39	Odds Ratio (IV, Random, 95% CI)	4.99 [1.27, 19.67]
4. HRQoL	1	982	619	63.03	Odds Ratio (IV, Random, 95% CI)	1.71 [1.50, 1.94]
5. HSQ	1	415	382	92.05	Odds Ratio (IV, Random, 95% CI)	11.58 [8.12, 16.51]
**Overall (UHC)**	20	36765	**16,770**	**45.61**	Odds Ratio (IV, Random, 95% CI)	1.92 [1.44, 2.58]

#### Two-population group (insured vs uninsured)

**Figure 5.**
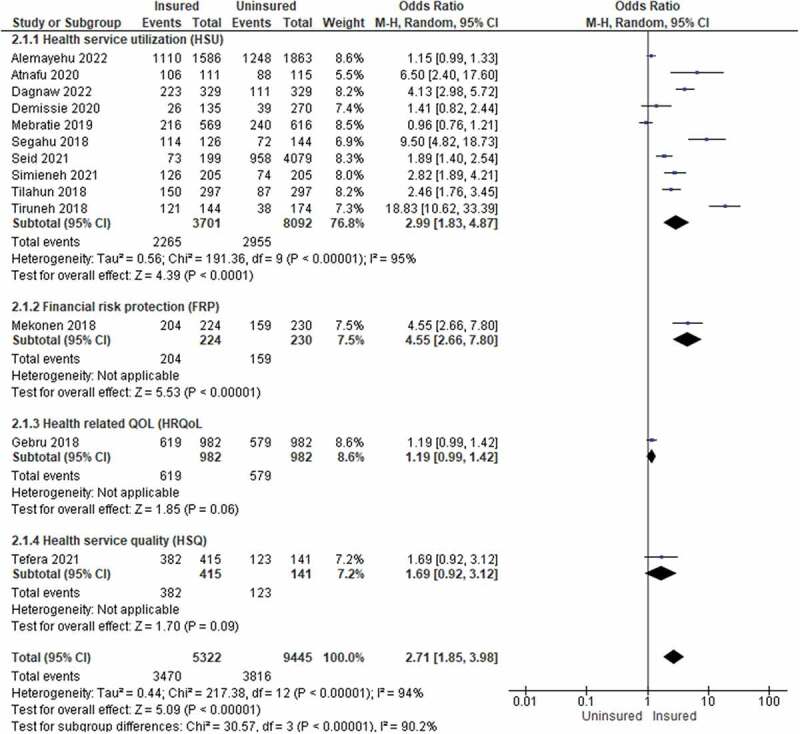
The forest plot for the two-population group (insured versus uninsured), Ethiopia, 2022.

The pooled result revealed that families with insurance had a 2.71-fold (OR = 2.71, 95% CI: 1.85–3.98) higher likelihood of UHC than households without insurance (Table 6 and Figure 5). CBHI users were 4.55 times (OR = 4.55, 95% CI: 2.66–7.80) more likely to be protected from CHE than non-users. The odds of HSU were found to be 2.99 times (OR = 2.99, 95% CI: 1.83–4.87) higher among families with insurance than those without it. Though it was not significant, families with insurance had a 1.69-times (OR = 1.69, 95% CI: 0.92–3.12) higher likelihood of having a perception of HSQ than households without insurance.

**Table 6.** The pooled result of the impact of CBHI on UHC for the two-population group (insured versus uninsured), Ethiopia (*n *= 13), 2022.

Outcome/Subgroup	Studies	Participants	Statistical Method	Effect Estimate
1. HSU	10	11793	Odds Ratio (M-H, Random, 95% CI)	2.99 [1.83, 4.87]
2. FRP	1	454	Odds Ratio (M-H, Random, 95% CI)	4.55 [2.66, 7.80]
3. HRQoL	1	1964	Odds Ratio (M-H, Random, 95% CI)	1.19 [0.99, 1.42]
4. HSQ	1	556	Odds Ratio (M-H, Random, 95% CI)	1.69 [0.92, 3.12]
**Overall (UHC)**	**13**	**14767**	**Odds Ratio (M-H, Random, 95% CI)**	**2.71 [1.85, 3.98]**

### Reporting biases

Most of the studies were conducted in the Amhara region (*n* = 12). After Amhara, most of them were nationwide (*n* = 7). The rest were conducted in Addis Ababa (*n* = 1), SNNPR (*n* = 2), and Oromia (*n* = 1). Due to the location bias of the reports, we did not conduct sub-group analyses based on region.

### Certainty of evidence

**Figure 6.**
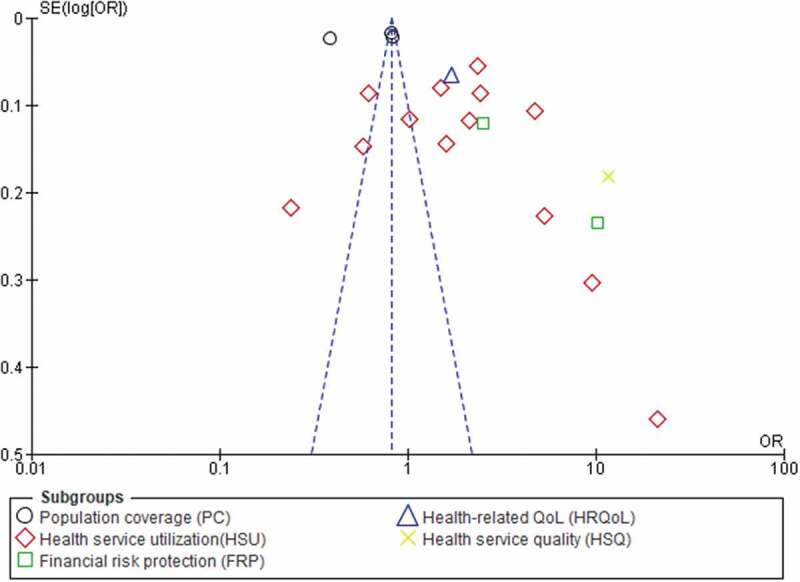
The funnel plot shows publication biases across the included studies.

The I^2^ statistic was used to evaluate between-study heterogeneity. For the one- and two-population groups, the I^2^ values were 99% and 94%, respectively, which are indicators of substantial heterogeneity [62]. Thus, since the I^2^ value was greater than 50%, a random-effects model was used to pool the impact of CBHI on UHC with a 95% CI [63]. The influence of each study on the overall meta-analysis was measured using IV. As portrayed in Figure 6, the funnel plots were used to examine the possibility of bias between studies (publication bias). Sensitivity analysis was performed by unchecking studies with small sample sizes (*n* < 200), but the heterogeneity remained the same. The I^2^ values across the sub-groups for the one- and two-population groups were 96.8% and 90.2%, respectively.

## Discussion

This review revealed that though the utilization of CBHI in Ethiopia had a significant impact on the step towards UHC, which was 20% by 2015 [64], the figure was still low (45.6%). However, ceteris paribus, being a CBHI member has increased the UHC by 24.8%, i.e. the UHC was 65.2% for the insured and 40.4% for the uninsured households. The pooled PC, HSU, FRP, HRQoL, and HSQ, respectively, were 40.1%, 64.5%, 79.4%, 63%, and 92.1%. A narrative review also revealed that health financing initiatives contributed to income generation, risk pooling, and the acquisition of healthcare services to support the road to UHC [65]. Other evidence in Africa and Asia also showed that health insurance has been found to have an impact on resource mobilization, FRP, service utilization, quality of care, social inclusion, and community empowerment [66].

**Figure 7.**
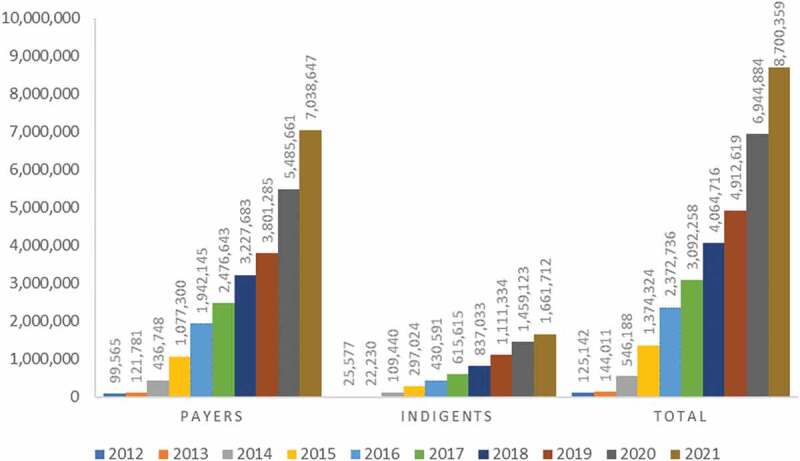
Enrollment trend of households in CBHI by year and payment modalities in Ethiopia [15,67].

However, according to this review, the PC was lower than the scheme’s national coverage in 2020, which was reported to be 50% [67]. Both the national report by the EHIS and the pooled result of this study were far below the national vision of reaching 80% of districts and 80% of the population by 2020 [68]. Though the main indicator of UHC is PC [69], the coverage was not consistent with the enrollment rate. According to the agency’s report, the number of households enrolled increased dramatically from 2012 through 2020 [67] and 2021 [15] (Figure 7). In 2021, functional districts had a total enrollment rate of 61% [15], which was higher than the enrollment rate of 44% in 2019 [14].

The imbalance between the enrollment and the coverage rate might be due to various reasons. First, voluntary membership gives families the freedom to join and leave as they wish based solely on their health status [67]. This can lead to adverse selection [70]. A high dropout rate from insurance schemes was also reported in Tanzania as a major challenge to UHC [71]. Since a CBHI plan is typically voluntary, without an adequate subsidy, poor households might not be interested in paying the premium, which leads to low participation and the exclusion of the poorest households [16]. As a result, households from the wealthiest subgroup in low- and middle-income nations were 61% more likely to enroll in health insurance than households from the poorest group in the same country [72]. The second possible reason could be the difference in the design characteristics of the scheme across regions. The CBHI members in SNNPR have limited access to tertiary health care services. In this region, insured households use tertiary services only at the nearest public hospitals, while those in Amhara may visit any public hospitals within the region, and those in Oromia may use care from public hospitals both within and outside the region. Insured households in SNNPR cannot claim reimbursements if they use health care services from private providers in the event that medical equipment or drugs are not available in CBHI-linked facilities [13]. Thirdly, even though the federal government’s general subsidy is set to be 25% of the total money to be collected, it has been found to have dropped to 10% since 2016, which may have a negative effect on the PC. The fourth reason may be poor targeted subsidization by the government. Except for SNNPR, the other regions have not fully covered the targeted subsidy (70% to be covered by the regional governments and 30% by districts) [67]. The fifth cause could be the settlement of agricultural households, which are widely dispersed and difficult to reach [70] but could be addressed by door-to-door (or hut-to-hut) outreach by insurance workers [73].

As a result, CBHI cannot be expected to provide a primary source of coverage to achieve UHC [16] unless critical measures are implemented, such as flexible payment plans that allow members to pay in installments, subsidized premiums for the poor, and the elimination of co-pays [74]. This is due to the fact that mandatory financial protection plans supported by general government funding that provide subsidies for those unable to pay have demonstrated a greater potential to achieve UHC than voluntary programs [16]. Since distinct pools for the subsidized maintain inequitable access, countries with indirect targeting or a universalist strategy have higher PC rates [75]. Total PC rates and the share of the subsidized in the total insured population could also be increased by broader eligibility criteria [76]. This is because greater health insurance coverage has been shown to improve health status, FRP, and access to healthcare facilities [77].

Though the PC is far below the plan, CBHI has significantly improved the HSU of the insured population in situations such as ANC and child healthcare visits, seeking treatment, in-patient and outpatient attendance, frequency of health facility visits, and family planning. This might be because, while visiting health facilities, the CBHI members could be informed about and become aware of exempted services like family planning and ANC services and be able to use those services [53]. Another review also found that health insurance improved the availability and delivery of maternal and neonatal health services and outcomes [78]. Similarly, in Vietnam, health insurance was found to improve access to and utilization of healthcare for the poor, children, and students [79]. This was also true in India, where children, pregnant women, and the poorest members of the insured population had increased their use of inpatient care as a result of health insurance [80]. It also improved the utilization of both outpatient and inpatient care among the insured elderly population in Tanzania [81]. CBHI improved households’ HSB from modern healthcare providers by reducing OOP payments [58]. By reducing per-capita health expenditure and increasing consumption per-capita, it improved household welfare [59]. In doing so, CBHI reduced health inequalities [82]. This review found that the HSU among the insured (61.2%) was approximately twice that of the uninsured (36.5%) households. Other studies also reported that health insurance improved HSU [66,83,84]. Thus, by improving HSU, CBHI was found to reduce mortality [85]. Nevertheless, insured non-poor households use more health care services than insured poor households, with a comparable effect on reducing health-related emergency expenditures [86].

The FRP, or reduction in exposure to CHE, was higher among insured households (91.1%) than uninsured households (69.1%). There is strong evidence that CBHI provides some FRP by reducing OOP spending. However, there is moderate evidence that such schemes improve cost recovery [87]. The positive impact of CBHI on FRP was also consistently reported by other studies [66,77,83,84]. Though health insurance schemes seemed to prevent CHE to a certain extent, reimbursement rates were reported to be very low, and vulnerable individuals often faced OOP payments [84]. It might be for such a reason that the OOP payment in Ethiopia is still the highest (34.4%) in Africa, only preceded by Ghana, where the OOP was 40% [88]. In fact, as a review in India shows, OOP expenditures are huge even after the FRP given by a number of health insurance programs [89]. Thus, the fundamental challenges to achieving UHC are not only spending more on health but also reducing the proportion of OOP spending. So it is important that more fiscal resources are needed to mitigate this [90]. OOP spending could be reduced by broadening the range of benefit packages, which would improve access to healthcare [75]. In particular, expanding pharmaceutical coverage may decrease overall OOP payments and unmet medical needs [91].

Though it was not found to be significant, the HSQ was also perceived to be a little higher among the insured (92.1%) than the non-insured households (87.2%). CBHI improved diagnostic test capacity, availability of tracer drugs, provider interpersonal communication, and service quality standards. The scheme also increased the accountability of health facilities in CBHI districts because they promised to provide quality services using the CBHI premium from enrolled households. As such, the scheme improved access to modern healthcare services. However, there was no strong evidence regarding the positive effect of CBHI on quality of care [66,83]. Being insured has not been linked to receiving higher-quality care [92]. Schemes that emphasize patients’ bargaining power at the patient-provider interface, however, appear to increase access to high-quality care [93]. In fact, Ethiopia’s CBHI had a considerable positive impact on healthcare infrastructures, medical supplies, diagnostic capacity, pharmaceuticals, FRP, and healthcare services [65]. However, the thrust of the service delivery process seems to be far behind. Shortages of drugs, frequent stockouts, prolonged reimbursement processes, overcrowding at public health facilities, the charging of unnecessary prices by private pharmacies to insurance beneficiaries, and confusion about annual renewal payments without using the service are all concomitant issues [94]. Low healthcare funding and high OOP payments contribute to limited access to equitable and high-quality healthcare services. These service discrepancies can be controlled through the standardization of benefit packages, ensuring beneficiaries have equal access to care, and establishing an accreditation system to uphold healthcare quality [65].

There was no significant difference between insured and uninsured families regarding HRQoL. However, the HRQoL among the insured households (63%) was slightly higher than that of those who were not insured (59%). There is some evidence that health insurance programs improve the health of insured households [77]. Though no significant difference was found, various patient groups without health insurance had lower mean HRQoL scores than those with health insurance [95,96].

### Limitations

Most of the included studies were conducted in the Amhara region. Hence, we did not perform sub-group analysis by region. There were inconsistencies in reports regarding factors associated with the components of UHC: PC, HSU, FRP, HSQ, and HRQoL. As a result, we did not consider the factors affecting UHC. The UHC data were pooled despite high heterogeneity. Articles published in languages other than English and those with a high risk of bias were excluded. For mixed studies, the risk of bias was assessed only from the perspective of the quantitative part. Moreover, no studies were found from the supply (provider) or insurer sides. Thus, the review reflects findings from the demand side.

### Practice and policy recommendations

SDG 3 aims to achieve UHC, which calls for equal access to healthcare for all people by promoting health and well-being at all ages [97]. All nations strive to improve their citizens’ access to quality and equitable health care and financial security [98]. An effort toward UHC is a long-term policy engagement that needs both technical and political expertise [99]. Health financing policy is an integral part of efforts to move towards UHC. On the other hand, health system reforms must particularly aim at improving coverage and the associated intermediate goals (efficiency, equity, transparency, and accountability) if health financing policy is to be in line with the pursuit of UHC. The unit of analysis for goals and objectives must be the population and health system as a whole. What matters is how a scheme effects population progress toward UHC, not how it impacts each of its individual participants. A focus on specific schemes alone is incompatible with a UHC strategy and may even be detrimental to it, especially in terms of equity. On the other hand, a scheme can advance toward UHC if it is fully oriented towards system-level goals and objectives. Thus, it is necessary to move policy and policy analysis from the scheme level to the system level [98]. To do so, four broad types of pooling reforms are recommended: shifting to compulsory coverage, merging different pools, cross-subsidization of pools, and harmonization across pools [100]. All these can help transform the CBHI model into a national scheme [16].

### Direction to future research

Future research aimed at investigating gaps or challenges from the supply, provider, insurer, and demand sides of CBHI implementation towards UHC is recommended.

## Conclusion

Ethiopia’s CBHI improved the HSU of beneficiaries by significantly reducing their exposure to CHE. The scheme increased HSQ and improved HRQoL through the utilization of quality health services. The UHC provided by the scheme was below 50%, although it was higher among members. The PC (40%) was below Ethiopia’s national plan, which aimed to cover 80% of districts and 80% of the population by 2020.

## Supplementary Material

Supplemental Material

## Paper context

Ethiopia has been implementing community-based health insurance since 2011 with the aim of achieving universal health coverage. However, the nationwide impact of the scheme has still not been evaluated comprehensively. Therefore, in order to address this, we undertook a systematic review and meta-analysis. It revealed that less than 50% of the population had access to universal health care. The finding indicates that Ethiopia should move to a broader national scheme that covers the entire population.
