# Supplementary material for: The impact of community-based health insurance on universal health coverage in Ethiopia: a systematic review and meta-analysis
Source: Glob Health Action. 2023 Mar 22;16(1):2189764. doi: 10.1080/16549716.2023.2189764 (PMC10035959; doi:10.1080/16549716.2023.2189764)
Supplement: Supplemental Material [file ZGHA_A_2189764_SM3731.zip › Supplementary_file_2._Database_sear_13.docx]

**Supplementary file 2. Database search strategy**

1. **PubMed (n = 40):**

Search: **(((((Impact) OR (Role)) OR (Effect)) AND (Community-Based Health Insurance)) AND (Ethiopia)) AND (("2012/01/01"[Date - Create] : "2022/09/27"[Date - Create]))**

("impact"[All Fields] OR "impactful"[All Fields] OR "impacting"[All Fields] OR "impacts"[All Fields] OR "tooth, impacted"[MeSH Terms] OR ("tooth"[All Fields] AND "impacted"[All Fields]) OR "impacted tooth"[All Fields] OR "impacted"[All Fields] OR ("role"[MeSH Terms] OR "role"[All Fields]) OR ("effect"[All Fields] OR "effecting"[All Fields] OR "effective"[All Fields] OR "effectively"[All Fields] OR "effectiveness"[All Fields] OR "effectivenesses"[All Fields] OR "effectives"[All Fields] OR "effectivities"[All Fields] OR "effectivity"[All Fields] OR "effects"[All Fields])) AND ("community based health insurance"[MeSH Terms] OR ("community based"[All Fields] AND "health"[All Fields] AND "insurance"[All Fields]) OR "community based health insurance"[All Fields] OR ("community"[All Fields] AND "based"[All Fields] AND "health"[All Fields] AND "insurance"[All Fields]) OR "community based health insurance"[All Fields]) AND ("ethiopia"[MeSH Terms] OR "ethiopia"[All Fields] OR "ethiopia s"[All Fields]) AND 2012/01/01:2022/09/27[Date - Create]

**Translations**

**Impact:** "impact"[All Fields] OR "impactful"[All Fields] OR "impacting"[All Fields] OR "impacts"[All Fields] OR "tooth, impacted"[MeSH Terms] OR ("tooth"[All Fields] AND "impacted"[All Fields]) OR "impacted tooth"[All Fields] OR "impacted"[All Fields]

**Role:** "role"[MeSH Terms] OR "role"[All Fields]

**Effect:** "effect"[All Fields] OR "effecting"[All Fields] OR "effective"[All Fields] OR "effectively"[All Fields] OR "effectiveness"[All Fields] OR "effectivenesses"[All Fields] OR "effectives"[All Fields] OR "effectivities"[All Fields] OR "effectivity"[All Fields] OR "effects"[All Fields]

**Community-Based Health Insurance:** "community-based health insurance"[MeSH Terms] OR ("community-based"[All Fields] AND "health"[All Fields] AND "insurance"[All Fields]) OR "community-based health insurance"[All Fields] OR ("community"[All Fields] AND "based"[All Fields] AND "health"[All Fields] AND "insurance"[All Fields]) OR "community based health insurance"[All Fields]

**Ethiopia:** "ethiopia"[MeSH Terms] OR "ethiopia"[All Fields] OR "ethiopia's"[All Fields]

1. **Research4Life (R4L) - Hinari (N= 31):**

**Search:** ((Impact) OR (Role) OR (Effect)) AND (Community Based Health Insurance) AND (Ethiopia)

Selected by:

**Date of publication:** from 2012/1/1 - 2022/08/27

**Content Type:** Any type

**Discipline:** Any type

**Language:** English

**Limit to:** Items with full text online

1. **Google scholar (n = 15)** **– using “Perish or Publish” software:**

**Impact OR Role OR Effect Community Based Health Insurance Ethiopia [title]**

*Publish or Perish 8.4.4041.8250 (basic report)
WinPosix (x64) edition, running on WinPosix 10.0.19042 (x64)*

**Search terms**

**Title words:** impact OR role OR effect community based health insurance Ethiopia
**Years:** all
**Other options:** include citations; include patents

**Data retrieval**

**Data source:** Google Scholar
**Search date:** 2022-08-27 17:47:43 +0300
**Cache date:** 2022-08-27 17:47:47 +0300
**Search result:** [0] No error

***Important:*** *This data source provides only abbreviated data. Any ellipses (... marks) shown in this report originate with the data source; they are NOT caused by subsequent processing in Publish or Perish.*

**Metrics**

**Reference date:** 2022-08-27 17:47:47 +0300
**Publication years:** 2012-2022
**Citation years:** 10 (2012-2022)
**Papers:** 15
**Citations:** 103
**Citations/year:** 10.30 (acc1=6, acc2=5, acc5=2, acc10=1, acc20=0)
**Citations/paper:** 6.87
**Authors/paper:** 2.27/2.0/1 (mean/median/mode)
**Age-weighted citation rate:** 32.70 (sqrt=5.72), 16.41/author
**Hirsch h-index:** 5 (a=4.12, m=0.50, 97 cites=94.2% coverage)
**Egghe g-index:** 10 (g/h=2.00, 103 cites=100.0% coverage)
**PoP hI,norm:** 5
**PoP hI,annual:** 0.50
**Fassin hA-index:** 3

**Results**

B Demissie, KG Negeri (2020) **Effect of community-based health insurance on utilization of outpatient health care services in Southern Ethiopia: a comparative cross-sectional study**. *Risk Management and Healthcare Policy*, ncbi.nlm.nih.gov, cited by 25 (12.50 per year)

AM Mekonen, MG Gebregziabher, AS Teferra (2018) **The effect of community based health insurance on catastrophic health expenditure in Northeast Ethiopia: A cross sectional study**. *PloS one*, journals.plos.org, cited by 38 (9.50 per year)

T Gebru, K Lentiro (2018) **The impact of community-based health insurance on health-related quality of life and associated factors in Ethiopia: a comparative cross-sectional study**. *Health and Quality of Life Outcomes*, hqlo.biomedcentral.com, doi:10.1186/s12955-018-0946-3, cited by 19 (4.75 per year)

EC Mussa, D Agegnehu, E Nshakira-Rukundo (2022) **… cash transfers on enrolment in community-based health insurance among female-headed households in south Gondar zone, Amhara region, Ethiopia**. *SSM-Population Health*, Elsevier

MM Simieneh, M Yitayal, AA Gelagay (2021) **Effect of community-based health insurance on healthcare-seeking behavior for childhood illnesses among rural mothers in Aneded District, East Gojjam …**. *Risk Management and …*, ncbi.nlm.nih.gov, cited by 2 (2.00 per year)

DM Asfaw, SM Shifaw, AA Belete, SB Aychiluhm (2022) **The Impact of Community-Based Health Insurance on Household's Welfare in Chilga District, Amhara Regional State, Ethiopia. Front**. *Public Health*, researchgate.net

MY Jembere (2018) **… of community based health insurance scheme on financial protection and healthcare seeking behavior of households in Tehuledere District, Northeast Ethiopia**. *Int J Health Econ Policy*, article.ijohep.org, cited by 2 (0.50 per year)

YK Alemayehu, E Dessie, G Medhin, N Birhanu, ... (2022) **The impact of community-based health insurance on health service utilization and financial risk protection in Ethiopia**., researchsquare.com

D Nair, K Tushune (2012) **Reducing Health Inequalities Role of Community Based Health Insurance Schemes; Evidence from India and Ethiopia**. *articles from the*, researchgate.net

S Alemayehu (2021) **The Effect of Community-Based Health Insurance on Health-Related Quality of Life and Associated Factors Among Hypertensive Patients in Public Health …**.

MY Jembere (2018) **Community based health insurance scheme as a new healthcare financing approach in rural ethiopia: role on access, use and quality of healthcare …**. *Family Med Med Sci Res*, cited by 9 (2.25 per year)

M Yismaw (2017) **Role of Community Based Health Insurance on Health Service Provision and Healthcare Seeking Behavior of Households in Rural Ethiopia: The Case of …**. *Addis Ababa University*, cited by 6 (1.20 per year)

G Kassie **The effect of community-based health insurance on modern FP utilization in Ethiopia**. *5th International Conference on FP (ICFP)*, cited by 2 (0.00 per year)

H Abay (2021) **The effect of social marketing strategies on community based health insurance enrolment in Lideta sub-city, Addis Ababa, Ethiopia**., Addis Ababa University

DM Asfaw, SM Shifaw, AA Belete, SB Aychiluhm **The Impact of Community Based Health Insurance (CBHI) on Household's Welfare in Chilga District, Amhara Regional State, Ethiopia**. *Frontiers in Public Health*, Frontiers

1. **Scopus (n = 10****) – using “perish or publish” software:**

**Impact OR Role OR Effect Community Based Health Insurance Ethiopia [title]**

*Publish or Perish 8.4.4041.8250 (basic report)
WinPosix (x64) edition, running on WinPosix 10.0.19042 (x64)*

**Search terms**

**Title words:** impact OR role OR effect community based health insurance Ethiopia
**Years:** all

**Data retrieval**

**Data source:** Scopus
**Search date:** 2022-08-27 17:48:43 +0300
**Cache date:** 2022-08-27 17:48:45 +0300
**Search result:** [0] No error

***Important:*** *This data source returns only one author per article; this affects the calculation of per-author metrics.*

**Metrics**

**Reference date:** 2022-08-27 17:48:45 +0300
**Publication years:** 2015-2022
**Citation years:** 7 (2015-2022)
**Papers:** 10
**Citations:** 98
**Citations/year:** 14.00 (acc1=7, acc2=7, acc5=3, acc10=0, acc20=0)
**Citations/paper:** 9.80
**Authors/paper:** 1.00/1.0/1 (mean/median/mode)
**Age-weighted citation rate:** 30.08 (sqrt=5.48), 30.08/author
**Hirsch h-index:** 5 (a=3.92, m=0.71, 91 cites=92.9% coverage)
**Egghe g-index:** 9 (g/h=1.80, 98 cites=100.0% coverage)
**PoP hI,norm:** 5
**PoP hI,annual:** 0.71
**Fassin hA-index:** 3

**Results**

A.M. Mekonen (2018) **The effect of community based health insurance on catastrophic health expenditure in Northeast Ethiopia: A cross sectional study**. *PLoS ONE* 13(10), ISSN 1932-6203, doi:10.1371/journal.pone.0205972, cited by 24 (6.00 per year)

A.D. Mebratie (2019) **The impact of Ethiopia's pilot community based health insurance scheme on healthcare utilization and cost of care**. *Social Science and Medicine* 220, pp. 112-119, ISSN 0277-9536, doi:10.1016/j.socscimed.2018.11.003, cited by 22 (7.33 per year)

Z. Yilma (2015) **Health risk and insurance: Impact of ethiopia's community based health insurance on household economic welfare**. *World Bank Economic Review* 29, ISSN 0258-6770, doi:10.1093/wber/lhv009, cited by 21 (3.00 per year)

B. Demissie (2020) **Effect of community-based health insurance on utilization of outpatient health care services in southern ethiopia: A comparative cross-sectional study**. *Risk Management and Healthcare Policy* 13, pp. 141-153, ISSN 1179-1594, doi:10.2147/RMHP.S215836, cited by 13 (6.50 per year)

T. Gebru (2018) **The impact of community-based health insurance on health-related quality of life and associated factors in Ethiopia: A comparative cross-sectional study**. *Health and Quality of Life Outcomes* 16(1), ISSN 1477-7525, doi:10.1186/s12955-018-0946-3, cited by 11 (2.75 per year)

Z. Shigute (2020) **The effect of ethiopia’s community-based health insurance scheme on revenues and quality of care**. *International Journal of Environmental Research and Public Health* 17(22), pp. 1-17, ISSN 1661-7827, doi:10.3390/ijerph17228558, cited by 5 (2.50 per year)

M.M. Simieneh (2021) **Effect of community-based health insurance on healthcare-seeking behavior for childhood illnesses among rural mothers in Aneded district, east Gojjam zone, Amhara region, northwest Ethiopia**. *Risk Management and Healthcare Policy* 14, pp. 1659-1668, ISSN 1179-1594, doi:10.2147/RMHP.S298658, cited by 2 (2.00 per year)

W.T. Bekele (2022) **Predictors of Community-Based Health Insurance in Ethiopia via Multilevel Mixed-Effects Modelling: Evidence from the 2019 Ethiopia Mini Demography and Health Survey**. *ClinicoEconomics and Outcomes Research* 14, pp. 547-562, ISSN 1178-6981, doi:10.2147/CEOR.S368925

D.M. Asfaw (2022) **The Impact of Community-Based Health Insurance on Household's Welfare in Chilga District, Amhara Regional State, Ethiopia**. *Frontiers in Public Health* 10, ISSN 2296-2565, doi:10.3389/fpubh.2022.868274

E.C. Mussa (2022) **Impact of conditional cash transfers on enrolment in community-based health insurance among female-headed households in south Gondar zone, Amhara region, Ethiopia**. *SSM - Population Health* 17, ISSN 2352-8273, doi:10.1016/j.ssmph.2022.101030

1. **Semantic Scholar (n = 20) – using “perish or publish” software:**

**Impact of Community Based Health Insurance in Ethiopia**

*Publish or Perish 8.4.4041.8250 (basic report)
WinPosix (x64) edition, running on WinPosix 10.0.19042 (x64)*

**Search terms**

**Keywords:** impact of community based health insurance in Ethiopia

**Data retrieval**

**Data source:** Semantic Scholar
**Search date:** 2022-08-27 18:59:16 +0300
**Cache date:** 2022-08-27 18:59:17 +0300
**Search result:** [0] No error

***Important:*** *The Semantic Scholar API is still under development and only provides limited paper search options and limited result data.*

**Metrics**

**Reference date:** 2022-08-27 18:59:17 +0300
**Publication years:** 2004-2022
**Citation years:** 18 (2004-2022)
**Papers:** 20
**Citations:** 326
**Citations/year:** 18.11 (acc1=11, acc2=8, acc5=5, acc10=1, acc20=0)
**Citations/paper:** 16.30
**Authors/paper:** 4.05/3.5/multi (mean/median/mode)
**Age-weighted citation rate:** 51.48 (sqrt=7.17), 15.62/author
**Hirsch h-index:** 8 (a=5.09, m=0.44, 311 cites=95.4% coverage)
**Egghe g-index:** 18 (g/h=2.25, 326 cites=100.0% coverage)
**PoP hI,norm:** 6
**PoP hI,annual:** 0.33
**Fassin hA-index:** 5

**Results**

Teklemichael Gebru, Kifle Lentiro (2018) **The impact of community-based health insurance on health-related quality of life and associated factors in Ethiopia: a comparative cross-sectional study**., doi:10.1186/s12955-018-0946-3, cited by 13 (3.25 per year)

Dagmawe Menelek Asfaw, Sirage Mohammed Shifaw, Atinkugn Assefa Belete, Setognal Birara Aychiluhm (2022) **The Impact of Community-Based Health Insurance on Household's Welfare in Chilga District, Amhara Regional State, Ethiopia**., doi:10.3389/fpubh.2022.868274

Lesanework Alemu Abenet, B. Alemu, Migbaru Alamirew (2019) **The Impact of Community Based Health Insurance Scheme on Health Care Utilization in North Achefer Woreda, West Gojjam Zone, Amhara Region, Ethiopia**., doi:10.32622/ijrat.711201935, cited by 1 (0.33 per year)

A. Mebratie, R. Sparrow, Z. Yilma, D. Abebaw, G. Alemu, A. Bedi (2019) **The impact of Ethiopia's pilot community based health insurance scheme on healthcare utilization and cost of care.**., doi:10.1016/j.socscimed.2018.11.003, cited by 38 (12.67 per year)

Essa Chanie Mussa, D. Agegnehu, Emmanuel Nshakira-Rukundo (2022) **Impact of conditional cash transfers on enrolment in community-based health insurance among female-headed households in south Gondar zone, Amhara region, Ethiopia**., doi:10.1016/j.ssmph.2022.101030

Z. Yilma, A. Mebratie, R. Sparrow, M. Dekker, G. Alemu, A. Bedi (2015) **Impact of Ethiopia’s Community Based Health Insurance on household economic welfare**., doi:10.1093/WBER/LHV009, cited by 59 (8.43 per year)

Z. Shigute, A. Mebratie, R. Sparrow, G. Alemu, A. Bedi (2020) **The Effect of Ethiopia’s Community-Based Health Insurance Scheme on Revenues and Quality of Care**., doi:10.3390/ijerph17228558, cited by 11 (5.50 per year)

Z. Yilma, A. Mebratie, R. Sparrow, M. Dekker, G. Alemu, A. Bedi (2015) **The impact of Ethiopia's community-based health insurance on household economic welfare : a policy brief**., cited by 1 (0.14 per year)

A. Mebratie (2015) **Essays on evaluating a community based health insurance scheme in rural Ethiopia**., cited by 11 (1.57 per year)

Molla Yismaw Jembere (2018) **Attitude of Rural Households towards Community Based Health Insurance in Northeast Ethiopia, the Case of Tehuledere District**., doi:10.4172/2167-1079.1000303, cited by 5 (1.25 per year)

A. Mebratie, R. Sparrow, Z. Yilma, D. Abebaw, G. Alemu, A. Bedi (2013) **Impact of Ethiopian pilot community-based health insurance scheme on health-care utilisation: a household panel data analysis**., doi:10.1016/S0140-6736(13)61346-X, cited by 47 (5.22 per year)

Z. Shigute, Christoph Strupat, F. Burchi, G. Alemu, A. Bedi (2019) **Linking Social Protection Schemes: The Joint Effects of a Public Works and a Health Insurance Programme in Ethiopia**., doi:10.1080/00220388.2018.1563682, cited by 10 (3.33 per year)

Bojia E. Duguma, T. Tesfaye, Asmamaw Kassaye, Anteneh Kassa, S. Blakeway (2021) **Control and Prevention of Epizootic Lymphangitis in Mules: An Integrated Community-Based Intervention, Bahir Dar, Ethiopia**., doi:10.3389/fvets.2021.648267

Y. Hailemichael, D. Hailemariam, K. Tirfessa, S. Docrat, A. Alem, G. Medhin, A. Fekadu, C. Lund, D. Chisholm, C. Hanlon (2021) **The Effect of Expanded Access to Mental Health Care on Economic Status of Households with a Person with a Mental Disorder in Rural Ethiopia: A Controlled Before-After Study**., doi:10.21203/rs.3.rs-1006902/v1

Oluwatosin O Adeyemo (2015) **Towards Universal Health Coverage in Nigeria: Can Community-Based Health Insurance Be Scaled Up?**.

A. Obse, J. Ataguba (2019) **Assessing medical impoverishment and associated factors in health care in Ethiopia**., doi:10.1186/s12914-020-00227-x, cited by 6 (2.00 per year)

Brook Legese, Boneya Gumi (2020) **Flooding in Ethiopia ; Causes , Impact , and Coping Mechanism . A Review**., cited by 2 (1.00 per year)

A. Asfaw, J. Braun (2004) **Is Consumption Insured against Illness? Evidence on Vulnerability of Households to Health Shocks in Rural Ethiopia**., doi:10.1086/423255, cited by 122 (6.78 per year)

Sintayehu Tilaye (2019) **The Current Practice of Corporate Social Responsibility in Ethiopia**., doi:10.5296/ijsw.v6i2.15567

Fábio Botelho, Karen Gripp, N. Yanchar, Abbie Naus, D. Poenaru, R. Baird, Eliane Reis, Leonildo Farias, Ana Gabriely Silva, Francisco Viana, José Armando Pessoa Neto, Sidney Silva, K. Ribeiro, L. Gatto, Matheus Daniel Faleiro, Miguel Godeiro Fernandez, Lucas S. Salgado, Natália Zaneti Sampaio, Matheus Daniel Faleiro, Anna Luiza Mendes, R. V. Ferreira, Luiz Marcião, Gabriel Canto, J. Borges, Victor Araújo, Gabriel Andrade, Joyce Braga, Lívia Bentes, Luís Pinto, Henry T Ndasi, L. Amlani, Ghislain Aminake, Xavier Penda, Serge Tima, A. Lechtig, Kiran J. Agarwal-Harding, Marta Whyte, M. Fowler-Woods, A. Fowler-Woods, G. Shingoose, Andrew R. Hatala, Felicia Daeninck, A. Vergis, K. Clouston, K. Hardy, Laure Djadje, O. M. Djoutsop, Adrien Tangmi Djabo, U. S. Kanmounye, Vanessa Nono Youmbi (2022) **Decolonizing Global Surgery**., doi:10.1503/cjs.007622
